# Dietary Supplementation with a 3-Selenoureidoindole Derivative Enhances Thermotolerance and Modifies the Hemolymph Amino Acid Profile in Silkworm (*Bombyx mori*)

**DOI:** 10.3390/biology15030245

**Published:** 2026-01-28

**Authors:** Yi Zhang, Xiaoning Sun, Meng Xu, Huan Liu, Shunyi Wang, Zhongjian Cai, Xinyue Guo, Shiqing Xu, Shunjun Ji, Yanghu Sima

**Affiliations:** 1 School of Life Sciences, Suzhou Medical College of Soochow University, Suzhou 215123, China; 20234221055@stu.suda.edu.cn (Y.Z.); xnsun@suda.edu.cn (X.S.); 20175221003@stu.suda.edu.cn (M.X.); 20185221004@stu.suda.edu.cn (X.G.); szsqxu@suda.edu.cn (S.X.); 2 College of Chemistry, Chemical Engineering and Materials Science, Soochow University, Suzhou 215123, China; liu930321@sina.cn (H.L.); shunyi@suda.edu.cn (S.W.); zjcai@suda.edu.cn (Z.C.); 3 Institute of Agricultural Biotechnology & Ecology (IABE), Soochow University, Suzhou 215123, China

**Keywords:** organic selenium, 3-selenoureidoindole derivative, *Bombyx mori*, thermotolerance, selenium fortification

## Abstract

The high bioavailability and low toxicity of organic selenium underscore its potential for nutritional fortification. This study investigated the biological effects of a novel 3-selenoureidoindole derivative (3-SeU-Ind) as a dietary selenium source in the invertebrate model organism silkworm (*Bombyx mori*). When reared on natural mulberry leaves, supplementation with 3-SeU-Ind (4–400 mg/L) had no significant effect on larval weight, pupal weight, or cocoon production performance. However, under compound diet conditions, the highest concentration (400 mg/L) significantly reduced both larval and pupal weights. Selenium was effectively accumulated in larval tissues and the pupal body. Under high-temperature stress, supplementation with 3-SeU-Ind (100 and 400 mg/L) significantly enhanced silkworm survival, which was associated with the upregulation of key antioxidant genes, including *MnSOD*, *CAT*, *GPX*, and *TrxR*. Furthermore, the supplementation altered methionine and lysine levels in the hemolymph in a sex-specific manner. Thus, 3-SeU-Ind demonstrated potential as a safe and effective selenium supplement.

## Simple Summary

Organic selenium supplements exhibit enhanced bioactivity and a broader safety dosage range compared to their inorganic counterparts, although their specific effects are contingent upon the compound’s structural composition. This study investigated the biological impacts of a novel organic selenium compound, specifically a 3-selenoureidoindole derivative, as a dietary selenium source in silkworms. The results indicated that selenium was effectively accumulated in the silkworms, leading to improved tolerance to high temperatures and an upregulation of antioxidant gene expression. Furthermore, the compound exerted sex-specific regulatory effects on methionine and lysine concentrations in the hemolymph. These findings contribute valuable insights into the development of organic selenium supplements.

## 1. Introduction

As an essential trace element for humans and other animals, selenium (Se) has an indispensable role in various physiological processes, including antioxidant defense, immune regulation, and metabolic balance [1,2]. Se-enriched agricultural and livestock production technologies, such as the cultivation of Se-rich crops, formulation of Se-enriched feeds, and microbial Se-rich fermentation, have advanced rapidly in recent years, providing diverse strategies for Se biofortification [3,4,5]. Currently, commonly available Se supplements are categorized into two main classes: inorganic Se (e.g., sodium selenite, Na_2_SeO_3_) and organic Se (e.g., selenomethionine, SeMet) [6]. Compared with its inorganic counterparts, organic Se generally exhibits higher bioavailability and lower cytotoxicity, making it the preferred form for nutritional and medical applications [7]. However, existing organic Se products (e.g., Se-enriched yeast, plant-derived bioactive Se, and Se nanoparticles) still face significant application bottlenecks, including high production costs, poor stability, and concerns over their long-term safety [8,9]. Consequently, the development of novel organic Se compounds and evaluation of their biological effects hold considerable scientific and practical significance.

Indole-isoselenourea compounds are a new type of organic Se-containing heterocycles that combine indole and isoselenourea structures, which confer notable biological functionalities to their molecular framework. Studies show that the indole scaffold, as a “dominant structure,” prevalent in natural bioactive molecules like tryptophan and serotonin, is valuable in drug design [10,11]. Chalcogen-substituted indole derivatives show potential in treating AIDS, cancer, heart disease, and biological allergies [12,13,14]. From a molecular structural standpoint, designing indole–selenourea compounds involves integrating the highly reactive C3 position of the indole scaffold with a Se atom, known for its dual nucleophilic and electrophilic properties and unique Lewis basicity. This structure provides several theoretical benefits: the Se atom adjusts the electron density of the indole ring through p-π conjugation, acts as a flexible “synthetic handle” for creating various derivatives via its dynamic C–Se bond, and serves as a directing group for remote C–H functionalization. Additionally, the Se atom participates in “chalcogen bonding” and other non-covalent interactions, potentially improving binding affinity and specificity to biological targets. In vitro studies show that these compounds exhibit significant anti-proliferative effects against SGC7901 and HT1080 tumor cells [15,16]. However, the physiological effects, metabolic patterns, and accumulation characteristics of this compound in animal models remain to be elucidated.

Current research on Se in animal nutrition mainly targets mammals and poultry, aiming to improve production performance, meat and egg quality, and antioxidant capacity through dietary supplementation [17,18]. Studies have also explored using Se-enriched plants or insects as Se sources for livestock feed [19,20]. Nonetheless, the physiological regulatory mechanisms of Se in insects themselves remain poorly explored. The silkworm, characterized by its brief life cycle and well-established genetic background, provides an advantageous model for evaluating the functional impacts of exogenous substances [21]. In the field of sericulture, high temperature represents a significant factor contributing to diminished silk production in the silkworm [22]. Exposure to heat stress adversely affects larval growth and development, impairs nutrient absorption, and modifies the composition of the intestinal microbiota [23,24]. Moreover, empirical evidence suggests that high temperatures can trigger autophagy in the midgut and silk gland tissues of silkworms through a calcium-mediated pathway, facilitating the progression from autophagy to apoptosis [25]. Se, an antioxidant trace element [26], may boost silkworm thermotolerance by influencing redox balance. The hemolymph is crucial for nutrient metabolism and transport, and changes in its amino acid profile affect the organism’s physiology [27]. However, the impact of organic Se on this profile is not yet studied. The silkworm is crucial to the sericulture industry and offers significant nutritional value, particularly its pupae, which are rich in crude protein with a balanced amino acid profile. About 30% of their composition is lipids, providing a good source of polyunsaturated fatty acids, including α-linolenic acid [28]. They also offer health benefits, including liver protection, immune support, and regulation of blood glucose and lipid levels [29].

This study used the silkworm as a model organism to investigate the biological effects of a novel indole–selenourea compound, and evaluated its impact on silkworm heat tolerance and hemolymph amino acid composition. The results provided theoretical support for the application of this novel Se source in insect Se nutrition research and the development of functional silkworm-derived products.

## 2. Materials and Methods

### 2.1. Preparation of Experimental Animals

The silkworm genetic strains *Dazao*, *J×H* (Jingsong × Haoyue), and *Huakang No. 2* used in this experiment were obtained from the Silkworm Germplasm Resource Bank of Soochow University, where *Dazao* is a classic experimental strain widely used in basic silkworm research, *J×H* is a spring strain commonly applied in sericulture production, and *Huakang No. 2* represents a summer strain with the largest rearing scale in the silk industry. After hatching, the larvae were reared at 26 ± 2 °C and 70–85% relative humidity under a 12 h light:12 h dark photoperiod, and fed fresh mulberry leaves three times daily. The *Guangshi No. 1* strain, an artificial-diet-adapted variety with high feeding performance, was kindly provided by the College of Forestry, Shandong Agricultural University. Larvae of this strain were reared on a compound feed under the following conditions: 27–30 °C and 80–90% relative humidity for the 1L (1st larva instar) to 3L, and 25–27 °C with 80–85% relative humidity for the 4L and 5L.

### 2.2. Dietary Supplementation of 3-SeU-Ind in Silkworms

The 3-SeU-Ind utilized in this investigation were synthesized and supplied by the College of Chemistry, Chemical Engineering, and Materials Science at Soochow University [16]. This compound has a molecular weight of 442.41 g/mol, comprises 17.85% selenium by mass, and demonstrates a purity exceeding 99.50%. Using 3 g/L γ-cyclodextrin as a cosolvent, the maximum solubilization concentration of the compound reached 400 mg/L. Aqueous solutions of 3-SeU-Ind were prepared at low (4 mg/L), medium (40 mg/L), and high (400 mg/L) concentration gradients, fully mixed, and ultrasonically treated for 1 h.

Dietary supplementation in mulberry leaf rearing: ddH_2_O, 3 g/L γ-cyclodextrin solution, and the respective organic Se solutions were evenly sprayed onto mulberry leaves at a ratio of 250 μL per gram (250 μL/g) of leaves. The leaves were air-dried at room temperature before being fed to silkworms. To account for potential losses during spraying, the following five groups were established based on the concentration of the sprayed solution: blank control group (CK), vehicle control group (Veh), low-concentration group (4 mg/L, 4), medium-concentration group (40 mg/L, 40), and high-concentration group (400 mg/L, 400). The expected content of 3-SeU-Ind in the treated leaves (*C*_leaf_, µg/g) was calculated using the following formula: $C_{leaf}=C_{soln}\times V_{add}$, where *C*_soln_ is the concentration of 3-SeU-Ind, and *V*_add_ is the volume of solution applied per unit mass of fresh leaves, which was 250 µL/g. Thus, the actual leaf contents for the groups sprayed with 4, 40, and 400 mg/L solutions were approximately 1 μg/g, 10 μg/g, and 100 μg/g, respectively. Larvae from the same batch (rearing box) were used for the experiment. All larvae were uniformly fed with CK group leaves in 1–4L. At 5L0h, larvae were sexed and allocated into groups. The larvae in each group were fed leaves corresponding to their respective treatment group until the wandering stage.

Dietary supplementation in compound diet rearing: Dry powdered compound diet (provided by Rudong County Sericulture Guidance Station, formulation shown in Table S1) was mixed with the above organic Se solutions at a concentration of 625 g/L, steamed for 30 min, and cooled before feeding. The experimental groups were set up identically to the mulberry leaf experiment, comprising five groups in total. The expected content of 3-SeU-Ind in the compound diet (*C*_diet_) was calculated using the following formula: $C_{diet}=\frac{C_{soln}}{m/V+\rho }$, where *C*_soln_ is the concentration of 3-SeU-Ind (mg/L), and *m*/*V* is the mixing ratio of dry diet to solution (625 g/L), and ρ is the density of the solution, approximated as that of water (1 kg/L). Based on this calculation, the actual contents of 3-SeU-Ind in the compound diets of each experimental group were approximately 2.47 μg/g, 24.70 μg/g, and 247.00 μg/g, respectively. Larvae from the same batch were used for the experiment. All larvae were uniformly fed with CK group compound diet from the 1–4L. At 5L0h, larvae were sexed and allocated into groups. The larvae in each group were fed leaves corresponding to their respective treatment group until the wandering stage.

### 2.3. Measurement of Larval Body Weight and Production Indicators

In both the mulberry leaf rearing and artificial diet rearing groups, following the grouping of larvae at the 5L0h stage, the total larval weight in each replicate rearing box was measured daily at a consistent time until the larvae reached the wandering stage. On the seventh day of the pupal stage, measurements of pupal weight and cocoon shell weight were taken, and the cocoon shell percentage was subsequently calculated.

For the determination of the sericin ratio, the dried cocoon shells were initially weighed (denoted as *G*_0_), cut into pieces, and immersed in a 25-fold volume of 0.2% sodium carbonate solution, followed by boiling for 30 min. The material was then rinsed three times with double-distilled water (ddH_2_O). This degumming procedure was repeated once. The degummed silk was subsequently dried in an oven at 90 °C for 6 h and weighed (denoted as *G*_1_). The sericin ratio (*SR*) was calculated using the formula: $SR=\frac{G_{0}-G_{1}}{G_{0}}$, where *G*_0_ represents the dry weight of the cocoon shell prior to degumming, and *G*_1_ represents the dry weight of the silk post-degumming.

### 2.4. Determination of Se Content

For the determination of Se content, samples were collected from various silkworm larval tissues, including the midgut, fat body, and silk gland, as well as from pupae, cocoon silk, and droppings. A 0.5 g portion of each tissue sample was weighed and subjected to pre-digestion with 6 mL of nitric acid for 12 h at room temperature. Subsequently, 2 mL of hydrogen peroxide was added, and the samples were digested using microwave heating. The digests were then cooled to below 60 °C and diluted to a final volume of 10 mL with 2% nitric acid. A standard curve was prepared by diluting a 1000 mg/L Se stock solution with 2% nitric acid to produce standards at concentrations of 5.0, 10.0, 20.0, 50.0, and 100.0 μg/L. All samples were analyzed using inductively coupled plasma mass spectrometry (ICP-MS) (Agilent, Santa Clara, CA, USA) employing a 1 μg/mL rhodium solution as the internal standard.

### 2.5. Hematoxylin-Eosin Staining

After 72 h of 3-SeU-Ind supplementation, five silkworm larvae were dissected from each group to collect midgut, silk gland, and fat body tissues. These tissues were rinsed three times in 0.7% physiological saline, fixed in 4% paraformaldehyde at 4 °C, dehydrated through a graded ethanol series, cleared in xylene, and embedded in paraffin wax. Sections were cut, mounted on glycerol-coated slides, baked at 50 °C for 6 h to enhance adhesion. Finally, the sections were stained with hematoxylin and eosin according to the manufacturer’s instructions. The tissue morphology was then examined and photographed under a microscope.

### 2.6. Assessment of Thermotolerance

Larvae from the same batch, reared on mulberry leaves, were utilized for the experiments. At 5L0h, the larvae underwent dietary supplementation with 3-SeU-Ind and were concurrently transferred to an incubator set at a temperature of 34 ± 1 °C to study the effects of elevated temperature, while maintaining constant light and humidity conditions. The experiment was conducted in three distinct batches. In Batch 1, larvae were not sexed, and five treatment groups were established: CK, Veh, 4, 40, and 400. Each group comprised 60 larvae, distributed across three boxes with 20 larvae per box. The survival rate of individuals in each group was recorded every 24 h until they reached the wandering stage. Based on the findings from Batch 1, Batch 2 included six treatment groups: CK, Veh, 50, 100, 200, and 300. Each group consisted of 90 larvae, distributed across three boxes with 30 larvae per box, and survival was recorded at 96 h. In Batch 3, larvae were sexed at 5L0h, and four treatment groups were established: Veh-Female, 100-Female, Veh-Male, and 100-Male. Each group comprised 90 larvae, distributed across three boxes with 30 larvae per box. The survival rate of individuals in each group was recorded every 12 h until they reached the wandering stage.

### 2.7. Quantitative Real-Time PCR (qRT-PCR)

After 3-SeU-Ind supplementation, larvae were dissected to collect midgut, silk gland, and fat body tissues. Total RNA was extracted with RNAiso Reagent (Takara, Dalian, China) and treated with DNase I to remove residual genomic DNA. First-strand cDNA was synthesized using a reverse transcription kit (Takara, Dalian, China). qPCR was conducted with SYBR Premix Ex Taq (TaKaRa, Dalian, China) in a 20 µL reaction volume, using *BmRp49* as an internal reference. Primer sequences are listed in Table S2. All reactions were performed on an ABI StepOnePlus™ Real-Time PCR System (Applied Biosystems, San Francisco, CA, USA).

### 2.8. Determination of Free Amino Acids in Silkworm Hemolymph

Under mulberry leaf rearing conditions, the *Huakang No. 2* strain was utilized for dietary supplementation with 3-SeU-Ind. Due to specific considerations in compound synthesis, a slightly modified version of 3-SeU-Ind was employed, with its molecular structure depicted in Figure S1. This compound has a molecular weight of 470.51 g/mol, comprises 16.78% selenium by mass, and demonstrates a purity exceeding 99.50%. Five experimental groups were established: CK, Veh, 4, 40, and 400. Hemolymph was collected by puncturing the larval caudal proleg with a sterile syringe and transferred into a tube pre-chilled on ice. It was then immediately diluted five-fold with ultrapure water, vortex-mixed, and centrifuged to obtain the supernatant. For derivatization, 200 µL of the supernatant or amino acid standard solution was placed in a 1.5 mL microcentrifuge tube, mixed with 20 µL of norleucine internal standard solution, followed by the addition of 200 µL of triethylamine-acetonitrile solution (to ensure pH > 7) and 100 µL of phenyl isothiocyanate-acetonitrile solution. After thorough mixing, the solution was incubated at 25 °C for 1 h. Then, 400 µL of n-hexane was added, and the mixture was shaken and allowed to stand for 10 min. The lower aqueous layer was collected and filtered through a 0.45 μm syringe filter prior to analysis.

Separation and detection were carried out using a L3000 high-performance liquid chromatograph (Rigol, Suzhou, China) equipped with a C18 reversed-phase column (250 mm × 4.6 mm, 5 μm) (Sepax, Newark, DE, USA). Mobile phase A was prepared by dissolving 7.6 g of anhydrous sodium acetate in 925 mL of ddH_2_O. The pH was adjusted to 6.5 using glacial acetic acid, followed by the addition of 70 mL of acetonitrile. The mixture was thoroughly mixed and filtered through a 0.45 μm membrane filter. Mobile phase B consisted of an 80% acetonitrile aqueous solution. Chromatographic conditions included an injection volume of 10 μL and a flow rate of 1.0 mL/min, column temperature 40 °C, run time 45 min. Calibration was performed using a series of amino acid standards processed identically to the samples, with the internal standard for quantification. Quality control included replicate injections of a pooled hemolymph sample and periodic analysis of calibration verifiers. The limits of detection and quantification were determined based on signal-to-noise ratios of 3:1 and 10:1, respectively.

### 2.9. Data Analysis

All statistical analyses were performed using Prism software (version 8.0, GraphPad Software, San Diego, CA). Statistical methods are detailed in Table 1. Differences among groups are indicated by different lowercase letters. The significance levels are denoted as follows: ^ns^, not significant; *, *p* < 0.05; **, *p* < 0.01; and ***, *p* < 0.001.

## 3. Results

### 3.1. Effects of Dietary Supplementation with 3-SeU-Ind on the Growth and Selenium Accumulation in Silkworms

**Figure 1:**
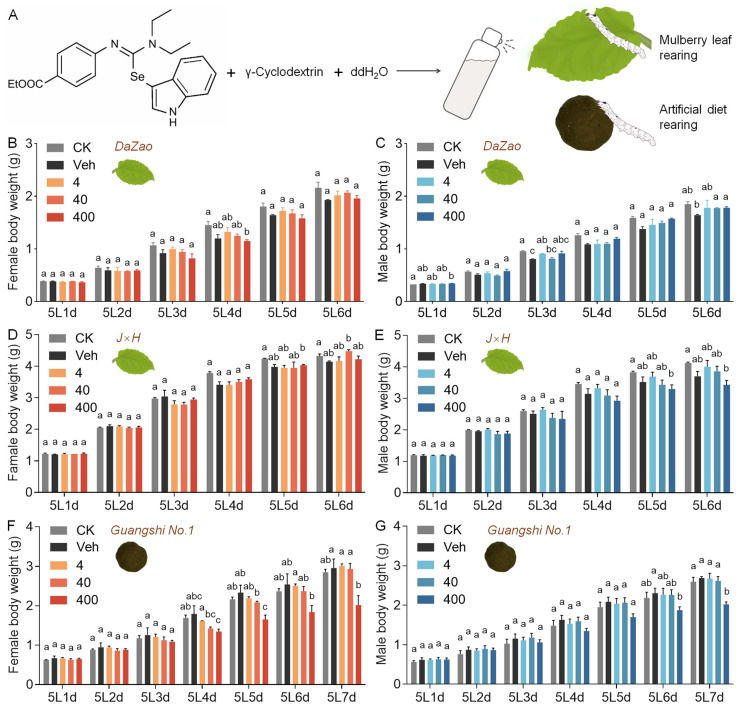
Impact of dietary 3-SeU-Ind supplementation on the body weight of male and female 5L silkworm larvae. (**A**) Molecular structure of 3-SeU-Ind and the experimental design for dietary supplementation in silkworms. (**B**–**G**) Changes in female (**B**,**D**,**F**) and male (**C**,**E**,**G**) body weight during the 5L larval stage of the (**B**,**C**) *Dazao*, (**D**,**E**) *J×H*, and (**F**,**G**) *Guangshi No. 1* strains fed either a natural mulberry leaf diet (**B**–**E**) or a compound feed (**F**,**G**). The treatment groups were as follows: CK, blank control group; Veh, vehicle control group; 4, 4 mg/L group (low concentration); 40, 40 mg/L group (medium concentration); 400, 400 mg/L group (high concentration). Data are mean ± SD; *n* = 3 biological replicates (3 individuals each) for (**B**–**G**). The different lower case letters show significant differences (*p* < 0.05).

**Figure 2:**
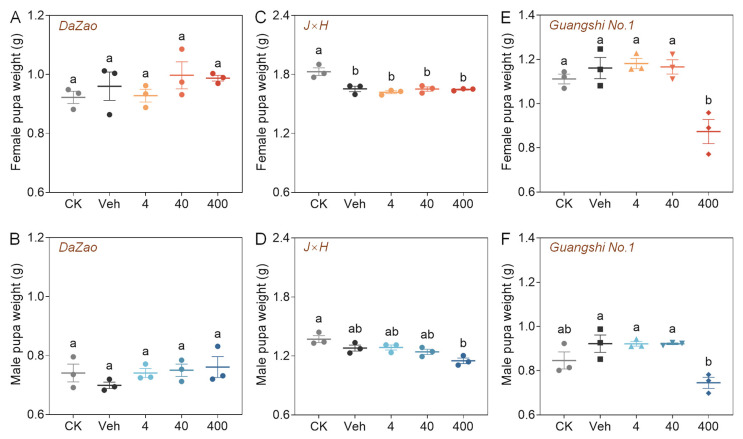
Effects of dietary 3-SeU-Ind supplementation on the pupal weights of different strains of male and female silkworms. Weights of female (**A**,**C**,**E**) and male (**B**,**D**,**F**) pupae from the (**A**,**B**) *Dazao*, (**C**,**D**) *J×H*, and (**E**,**F**) *Guangshi No. 1* strains of silkworm. Data are mean ± SD; *n* = 3 biological replicates (10–15 individuals each) for (**A**–**F**). The different lower case letters show significant differences (*p* < 0.05).

The effects of 3-SeU-Ind dietary supplementation were investigated on 5L of silkworms receiving either a natural mulberry leaf diet or a compound feed in five treatment groups (Figure 1A). Under a mulberry leaf diet, there was no significant difference in body weight in early-stage 5L larvae of the classical experimental strain *Dazao* and the cocoon-producing strain *J×H* among all five groups. In contrast, in late-stage 5L larvae of either strain, supplementation with low (4 mg/L) or medium (40 mg/L) concentrations of 3-SeU-Ind partially mitigated the negative effects on larval growth seen in the Veh (Figure 1B–E). Under a compound feed, supplementation with low or medium concentrations of 3-SeU-Ind did not significantly affect the body weight of 5L larvae of the *Guangshi No. 1* strain. In contrast, high-concentration supplementation caused a significant decrease in larval body weight from Days 4 to 6 of the 5L stage (Figure 1F,G). There was no significant difference in male and female pupal weights in the *Dazao* and *J×H* strains compared with the Veh following 3-SeU-Ind supplementation at any concentration (Figure 2A–D). Conversely, high-concentration supplementation led to decreased weights of both *Guangshi No. 1* female and male pupae (Figure 2E,F).

Under high-concentration supplementation, the sericin ratio in the silk glands of *Dazao* larvae was significantly lower than that in the Veh (Figure S2A). The qRT-PCR results also revealed significantly downregulated expression of the sericin genes *Ser1* and *Ser3* in silk gland tissues (Figure S2B–G). Assessment of cocoon production performance revealed no significant difference in cocoon shell weight or cocoon shell ratio for female and male *Dazao* across all supplementation groups, compared with the Veh (Figure S3). For the *J×H* strain, although female cocoon shell weight and the cocoon shell ratio for both sexes showed no significant difference among groups, the male cocoon shell weight in the high-concentration group was significantly lower than that in the Veh (Figure S4). Under high-supplementation conditions, both male and female shell weight and cocoon shell ratio of *Guangshi No. 1* pupae were significantly lower compared with the Veh (Figure S5). In conclusion, dietary supplementation with low and medium concentrations of 3-SeU-Ind compound did not significantly affect the growth or silk production capacity of silkworms under either rearing model.

**Figure 3:**
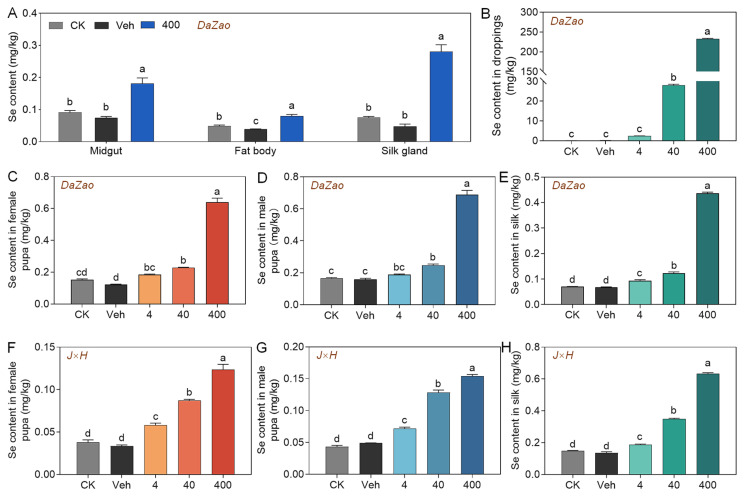
Distribution and accumulation of Se. (**A**) Se content in the midgut, fat body, and silk glands of the *Dazao* strain. (**B**) Se content in the droppings of the *Dazao* strain. (**C**,**D**) Se content in female (**C**) and male (**D**) pupae of the *Dazao* strain. (**E**) Se content in the silk cocoons of the *Dazao* strain. (**F**,**G**) Se content in female (**F**) and male (**G**) pupae of the *J×H* strain. (**H**) Se content in the silk cocoons of the *J×H* strain. Data are mean ± SD; *n* = 3 biological replicates (3 individuals each) for (**A**,**C**–**H**); *n* = 3 biological replicates (10–15 individuals each) for (**B**). The different lower case letters show significant differences (*p* < 0.05).

To investigate the accumulation of Se in silkworm, we determined the Se content in various tissues and silk cocoons. Se levels in the midgut, fat body, and silk gland of 5L larvae in the high-concentration supplementation group were significantly higher than those in the Veh, with the highest accumulation observed in silk glands (0.28 mg/kg) (Figure 3A). Hematoxylin and eosin staining revealed no apparent morphological change in these three tissues following supplementation with different concentrations of 3-SeU-Ind (Figure S6). Se content in larval droppings increased with supplementation concentration, peaking at 232.36 mg/kg in the *Dazao* strain and 310.03 mg/kg in the *J×H* strain (Figure 3B; Figure S7A). The Se content in female and male pupae as well as cocoon silk from the *Dazao* strain significantly increased at higher concentrations of 3-SeU-Ind (Figure 3C–E), with the *J×H* strain exhibiting a similar trend (Figure 3F–H). However, the transfer efficiency of Se to the silk glands gradually decreased at higher concentrations of 3-SeU-Ind (Figure S7B,C). These results demonstrated that dietary supplementation with 3-SeU-Ind effectively promoted Se accumulation in multiple tissues and silk cocoons of silkworm.

### 3.2. Dietary Supplementation with 3-SeU-Ind Enhances High-Temperature Tolerance in Silkworm

**Figure 4:**
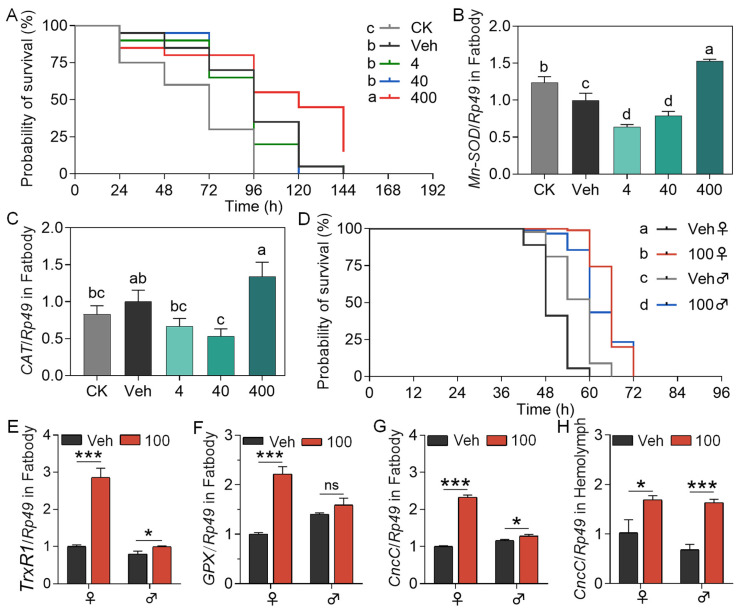
Effects of dietary 3-SeU-Ind supplementation on different metrics of the thermotolerance of 5L *Dazao* larvae exposed to 34 °C heat stress. (**A**) Percentage survival over time and (**B**,**C**) changes in the expression of (**B**) *MnSOD* and (**C**) catalase (*CAT*) in the fat body. The different lower case letters show significant differences (*p* < 0.05), analyzed with Survival analysis. (**D**) Percentage survival over time and (**E**–**H**) changes in the expression of (**E**) *TrxR1*, (**F**) *GPX*, and (**G**) *CncC* in the fat body and (**H**) *CncC* in the hemolymph in the 100 mg/L 3-SeU-Ind treatment group versus Veh. Data are mean ± SD; *n* = 60 individuals for (**A**); *n* = 3 biological replicates (3 individuals each) for (**B**,**C**); *n* = 60 individuals for (**D**); *n* = 3 biological replicates (3 individuals each) for (**E**–**H**). The different lower case letters show significant differences (*p* < 0.05); ^ns^, not significant (*p* > 0.05); * *p* < 0.05; *** *p* < 0.001.

High-temperature stress experiments revealed that dietary supplementation with 3-SeU-Ind at a concentration of 400 mg/L significantly improved the survival of *Dazao* larval fed mulberry leaf diet (Figure 4A). At this concentration, manganese superoxide dismutase (*MnSOD*) expression levels in the fat body were significantly upregulated compared with the Veh (Figure 4B), while catalase (*CAT*) expression also increased compared with the Veh, although the difference was not statistically significant (Figure 4C). Based on the aforementioned findings, six groups were established to receive supplementation with 3-SeU-Ind: CK, Veh, 50 mg/L, 100 mg/L, 200 mg/L, and 300 mg/L. After a 96 h exposure to high temperatures, survival rate analysis indicated that the 100 mg/L group demonstrated the highest survival rate, which was significantly greater than that of the Veh (Figure S8). Therefore, a concentration of 100 mg/L was chosen for subsequent 3-SeU-Ind supplementation studies. Results at this concentration revealed that survival curves for both male and female larvae under high temperature stress significantly differed from the Veh, indicating superior heat tolerance in the supplemented groups compared with Veh, with a more pronounced effect observed in females (Figure 4D). Gene expression analysis revealed that thioredoxin reductase (*TrxR*), involved in intracellular redox balance, was significantly upregulated in both females and males (Figure 4E). Glutathione peroxidase (*GPX*) expression was significantly upregulated in females treated with 100 mg/L compared with the Veh (Figure 4F). Concurrently, expression levels of Cap’n’collar C (*CncC*, the direct homolog of Nrf2) in the fat body and hemolymph of both female and male larvae were significantly higher than in the Veh (Figure 4G,H). Thus, 3-SeU-Ind enhanced the tolerance of silkworm to high temperatures by regulating the expression of antioxidant and related stress genes.

### 3.3. Dietary Supplementation with 3-SeU-Ind Alters the Hemolymph Amino Acid Profile in Silkworms

**Figure 5:**
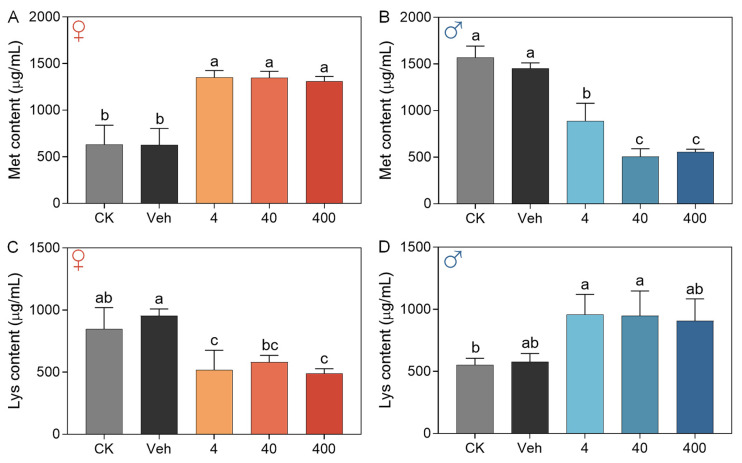
Effects of dietary 3-SeU-Ind supplementation on the amino acid composition of hemolymph in 5L larvae of *Huakang No. 2* silkworm. (**A**,**B**) Methionine (Met) content in the hemolymph of female (**A**) and male (**B**) larvae. (**C**,**D**) Lysine (Lys) content in the hemolymph of female (**C**) and male (**D**) larvae. Data are mean ± SD; *n* = 3 biological replicates (3 individuals each) for (**A**–**D**). The different lower case letters show significant differences (*p* < 0.05).

To augment its potential for application, 3-SeU-Ind underwent minor modifications and was subsequently assessed via dietary supplementation in both the *Dazao* strain and the summer-practical strain *Huakang No. 2* (Figure S9A). No significant differences were observed in the body weight of larvae or in pupal (both females and males) weight compared with the Veh in either strain (Figure S9B–I). These findings suggest that the modified compound maintains efficacy comparable to that of the original 3-SeU-Ind. To further evaluate the metabolic impact of 3-SeU-Ind, we analyzed the amino acid composition of the hemolymph of 5L larvae of the *Huakang No. 2* strain. Dietary supplementation with 3-SeU-Ind for 48 h altered the levels of multiple amino acids in the hemolymph, with some changes exhibiting distinct sexual dimorphism across five treatment groups (Table S3). The concentration of methionine (Met) increased significantly in female larvae compared with the Veh (Figure 5A), whereas it was significantly decreased in male individuals (Figure 5B). Conversely, 3-SeU-Ind treatment significantly reduced the lysine (Lys) level in the hemolymph of females compared with the Veh (Figure 5C), while there was an increase observed in males (Figure 5D). These results indicated that dietary 3-SeU-Ind supplementation could remodel the hemolymph amino acid profile in a sex-specific manner.

## 4. Discussion

This study examines the novel organic Se form, 3-SeU-Ind, to understand its biosafety effects in the silkworm, aiming to systematically elucidate its biosafety and physiological functions. Organic Se is typically more bioavailable and less toxic than inorganic Se, a difference potentially attributable to their distinct absorption mechanisms. Inorganic selenate is absorbed via hydrogen ion exchange and sodium pumps, while selenite enters the body through free diffusion. In contrast, organic Se compounds, such as selenoproteins and Se-polysaccharides, are digested into small molecules and absorbed primarily through amino acid transport pathways [30]. In livestock production, daily supplementation with 1.83 mg of organic Se (SeMet) significantly enhances ram semen quality compared to 4.0 mg of inorganic Se (sodium selenite) [31]. Similarly, hens consuming Se-enriched insect protein show better growth and higher egg Se content than those on Se-yeast diets [20]. Different organic Se compounds exhibit distinct functions; for example, synthetic diselenides have stronger antioxidant properties than monoselenides [32]. Common organic Se supplements, such as Se-enriched yeast, L-SeMet, and Se polysaccharides, are considered to hold greater application potential because they can be efficiently metabolized into SeCys and SeMet and subsequently incorporated into selenoproteins [33]. Therefore, developing new Se supplements with high bioavailability is essential for enhancing Se-fortified foods, biofortification strategies, and addressing human Se deficiency in human populations.

The mulberry leaves soaked in 50 μM Na_2_SeO_3_ solution for the diet can promote the growth, silk production and reproductive rate of silkworms, while at 200 μM concentration, it shows inhibitory effects [34]. However, our findings show that low-to-medium concentrations of the 3-SeU-Ind caused no significant effects on the growth, development, or key economic traits of silkworms. Furthermore, Se is effectively accumulated in the bodies of silkworms. These results support using 3-SeU-Ind as a new organic Se supplement and for creating functional silkworm products.

Se is a key antioxidant in organisms, with established roles in animals, plants, and fungi [35,36]. Selenoproteins mainly regulate redox homeostasis, modulating transcription factors, and orchestrating signaling pathways related to the antioxidant response element (ARE). In mammals, Se-dependent *GPXs* and *TrxRs* directly counteract oxidative stress [26]. Among them, GPX1 plays a central role in maintaining redox balance by scavenging reactive oxygen species (ROS) [37]. This study found that 3-SeU-Ind supplementation in silkworms significantly upregulates the expression of genes for *Mn-SOD*, *CAT*, *GPX*, and *TrxR*, along with a notable rise in *cncC* expression in the fat body and hemolymph. These findings are consistent with related reports: SeMet treatment upregulated *CAT* and *SOD1* expression in mouse liver [38]; Se-rich foods enhanced *GPX* and *CAT* activities, thereby improving male fertility [2]; and organic Se alleviated hypothalamic inflammation and autophagy dysregulation in pigs via the Keap1/Nrf2 axis [39]. Collectively, the 3-SeU-Ind enhances the antioxidant capacity of silkworms by activating a network of antioxidant gene expression, thus improving their thermotolerance.

The study revealed that 3-SeU-Ind supplementation significantly alters the hemolymph amino acid profile, showing notable sex-specific differences. Previous studies have shown that organic Se affects amino acid metabolism in animals, for instance, hydroxyl-selenomethionine supplementation in *Oncorhynchus mykiss* fry increased Cysteine and Cysteinylglycine concentrations while decreasing the levels of some essential amino acids in muscle [40], and organic Se supplementation in dairy goats was shown to intervene in blood protein metabolism processes [41], our findings provide new insights into its sex-dimorphic effects in insects. However, relevant research in insects remains scarce. After 48 h of treatment with the 3-SeU-Ind, significant sex-specific differences were observed in the levels of Met and Lys in the hemolymph. We speculate that this phenomenon may be linked to the differential involvement of 3-SeU-Ind in antioxidant defense and silk protein synthesis. In contrast, female individuals, under Se-induced oxidative stress response, may mobilize Lys more extensively for the synthesis of antioxidant-related products, leading to the observed sexual dimorphism in hemolymph Lys levels, while male silkworms might preferentially allocate Lys for silk protein synthesis. This finding not only expands our understanding of Se’s biological functions but also provides new experimental evidence for the interaction between trace elements and amino acid metabolism in insects, the deeper mechanisms of which warrant further exploration.

## 5. Conclusions

The research assessed the impact of 3-SeU-Ind as a source of organic Se on the biological parameters of silkworms. The findings demonstrated that varying concentrations of 3-SeU-Ind did not affect larval weight, pupal weight, or cocoon production performance in different silkworm strains under natural mulberry leaf rearing conditions. Supplementation with 3-SeU-Ind resulted in a significant accumulation of selenium across diverse tissues, pupae, and silk cocoons. Furthermore, it improved the thermotolerance of silkworms, which was associated with the upregulation of antioxidant-related genes, such as *MnSOD* and *TrxR*. Additionally, it induced sex-specific alterations in the metabolic levels of Met and Lys in the hemolymph. In conclusion, 3-SeU-Ind demonstrates the potential to be a safe and effective organic Se dietary supplement.

## Figures and Tables

**Table 1:** Summary of Statistical Methods.

Measurement	Primary Statistical Test	Post Hoc Comparisons	Adjustment for Multiple Testing	Assumptions Diagnostics	Defined Experimental Unit
Larval Body Weight	Two-way repeated measures ANOVA (Between-subjects factor: Treatment group; Within-subjects (repeated) factor: Time)	If the interaction was significant, pairwise comparisons between all treatment groups were performed at each time point.	Tukey’s HSD	1. Normality (Shapiro–Wilk test on residuals) 2. Homogeneity of variances (Mauchly’s test)	Each independent replicate rearing box. The value analyzed was the mean weight of all larvae within a box. *n* = 3 boxes per treatment group.
Pupa weight; Cocoon shell weight; Cocoon shell percentage; Survival rate	One-way ANOVA	If the overall test was significant, pairwise comparisons between all treatment groups were performed.	Tukey’s HSD	1. Normality (Shapiro–Wilk test) 2. Homogeneity of variances (Brown–Forsythe test)	*n* = 3 independent biological replicates per treatment group. Each replicate was represented by data obtained from three or more individual specimens, and the mean value was used as a single data point for analysis.
Sericin ratio; silk protein gene expression; Se content; Se transfer efficiency; *Mn-SOD* and *CAT* gene expression; Amino Acid content	One-way ANOVA	If the overall test was significant, pairwise comparisons between all treatment groups were performed.	Tukey’s HSD	1. Normality (Shapiro–Wilk test) 2. Homogeneity of variances (Brown–Forsythe test)	*n* = 3 independent biological replicates per treatment group. Each replicate was represented by a sample pooled from three or more individual larvae, and the measurement from this pooled sample was treated as a single data point.
Probability of survival	Kaplan–Meier survival analysis, with between-group comparisons using the Log-rank test	If the overall test was significant, pairwise comparisons between all treatment groups were performed.	Bonferroni correction	1. Independence: Death events for individual subjects are independent. 2. Non-informative censoring: The reason for a subject being censored (e.g., alive at the end of the study) is unrelated to their subsequent risk of death.	The individual larva. The analysis was based on the survival time of each individual.
*TrxR1*, *GPX*, and *CncC* gene expression	Independent samples T-test (for comparisons between two groups)	Not applicable	Not applicable	1. Normality (Shapiro–Wilk test) 2. Homogeneity of variances (F-test)	*n* = 3 independent biological replicates per treatment group. Each replicate consisted of tissue pooled from three or more individual larvae.

Note: The significance level used was α = 0.05.

## Data Availability

The original contributions presented in this study are included in the article/Supplementary Materials. Further inquiries can be directed to the corresponding authors.

## Supplementary Materials

The following supporting information can be downloaded at: https://www.mdpi.com/article/10.3390/biology15030245/s1, Figure S1: Schematic structure of the modified 3-selenoureidoindole derivative; Figure S2: Effects of dietary 3-SeU-Ind supplementation on the sericin ratio and silk protein gene expression in the *Dazao* strain; Figure S3: Effects of dietary 3-SeU-Ind supplementation on the cocoon production performance of the *Dazao* strain; Figure S4: Effects of dietary 3-SeU-Ind supplementation on cocoon production traits in the *J×H* strain; Figure S5: Effects of dietary 3-SeU-Ind supplementation on cocoon production traits in the *Guangshi No. 1* silkworm strain; Figure S6: Effects of dietary 3-SeU-Ind supplementation on the midgut, silk gland, and fat body tissues of the *Dazao* strain; Figure S7: Se content in silkworm droppings and its transfer efficiency to silk following dietary 3-SeU-Ind supplementation; Figure S8: Effects of dietary 3-SeU-Ind supplementation on the survival rate of 5L *Dazao* larvae after 96 h of 34℃ heat stress; Figure S9: Impact of dietary supplementation with slightly modified 3-SeU-Ind on the body weight of male and female 5L silkworm larvae; Table S1: Formula for compound feed for silkworms; Table S2: Primer Sequences; Table S3: Concentrations of various amino acids in hemolymph at 48 h after dietary 3-SeU-Ind supplementation.

## Abbreviations

The following abbreviations are used in this manuscript:

3-SeU-Ind	3-selenoureidoindole derivative
Se	Selenium
