# Supplementary material for: Dietary Supplementation with a 3-Selenoureidoindole Derivative Enhances Thermotolerance and Modifies the Hemolymph Amino Acid Profile in Silkworm (Bombyx mori)
Source: Biology (Basel). 2026 Jan 28;15(3):245. doi: 10.3390/biology15030245 (PMC12897462; doi:10.3390/biology15030245)
Supplement: Supplementary file 1 [file biology-15-00245-s001.zip › biology-4089891-supplementary.pdf]

biology

MDPI

Dietary Supplementation with a 3-Selenoureidoindole Derivative Enhances Thermotolerance and Modifies the Hemolymph Amino Acid Profile in Silkworm (*Bombyx mori*)

Supplementary Materials

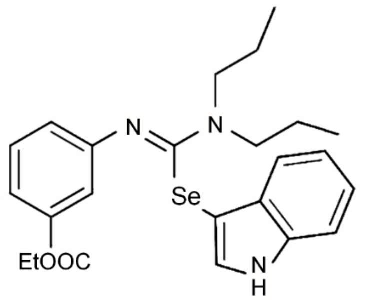

Figure S1. Schematic structure of the modified 3-selenoureidoindole derivative.

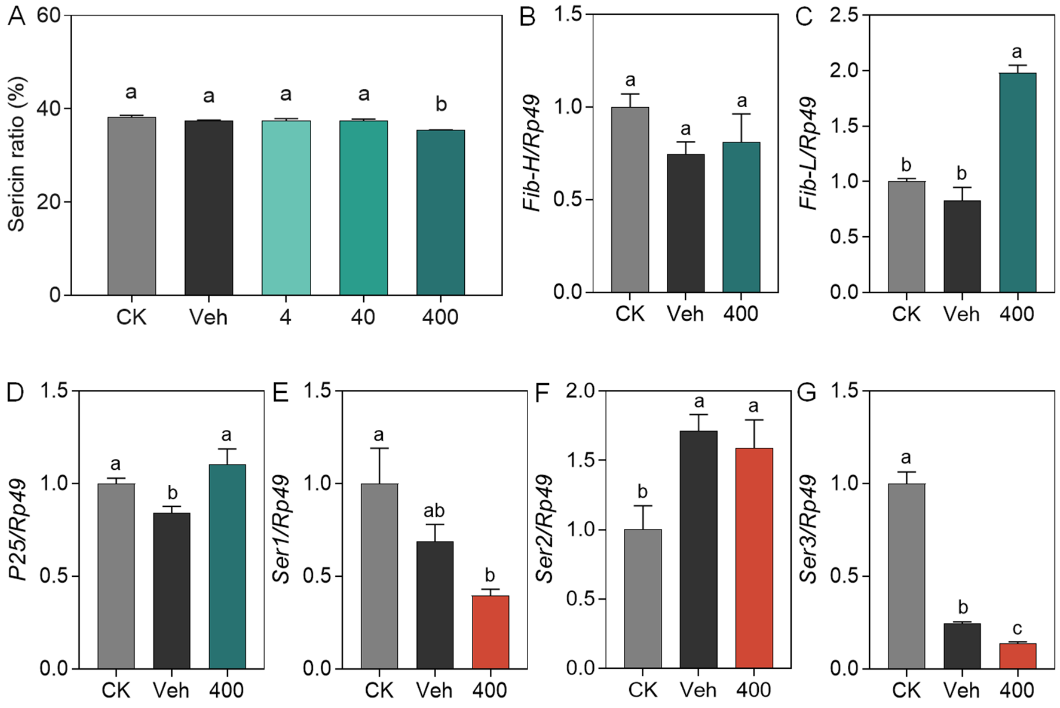

Figure S2. Effects of dietary 3-SeU-Ind supplementation on the sericin ratio and silk protein gene expression in the *Dazao* strain. (A) Sericin ratio. (B–G) Relative expression levels of silk protein genes. Data are mean ± SD; n =3 biological replicates (3 individuals each) for (A–G). The different lower case letters show significant differences ($P < 0.05$).

 https://doi.org/10.3390/biology15030245

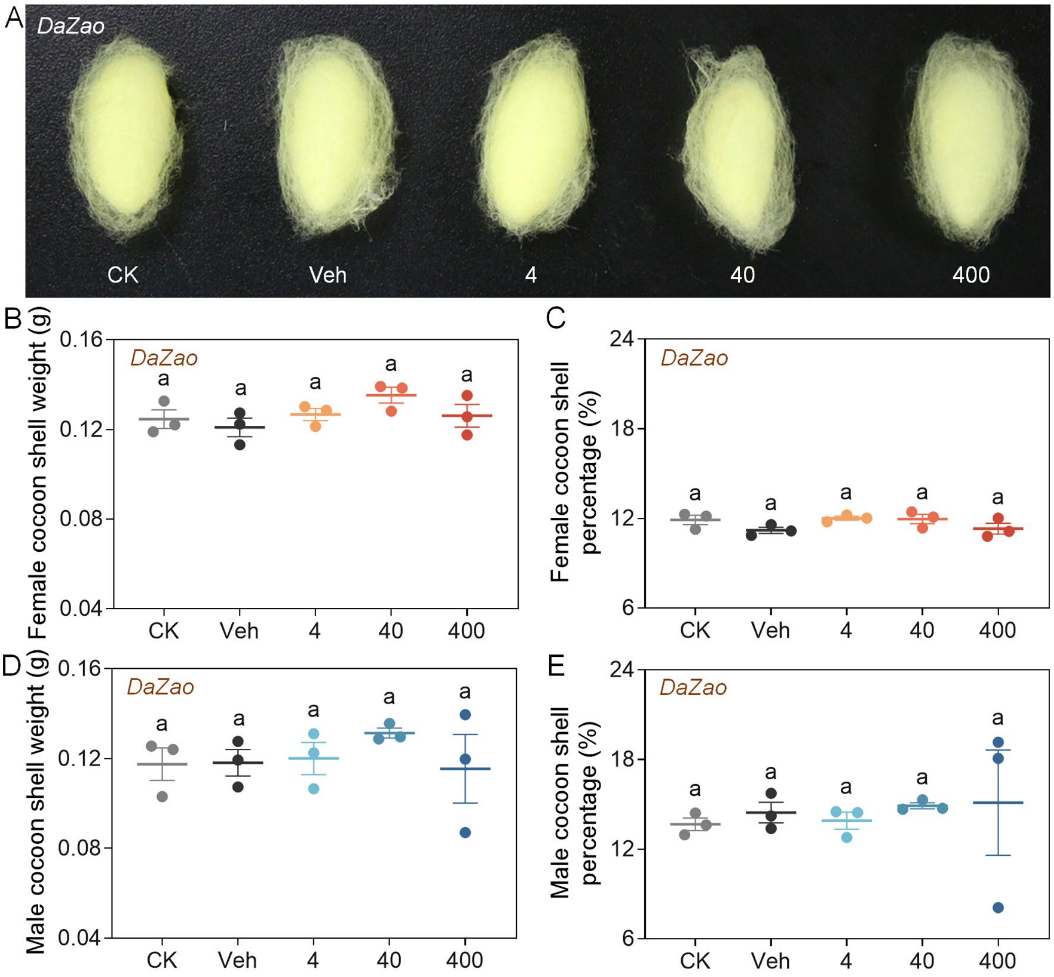

Figure S3. Effects of dietary 3-SeU-Ind supplementation on the cocoon production performance of the *Dazao* strain. (A) Representative images of cocoon morphology from each treatment group. (B,C) Cocoon shell weight (B) and cocoon shell ratio (C) of females. (D,E) Cocoon shell weight (D) and cocoon shell ratio (E) of males. Data are mean ± SD; *n* =3 biological replicates (15 individuals each) for (B–E). The different lower case letters show significant differences ($P < 0.05$).

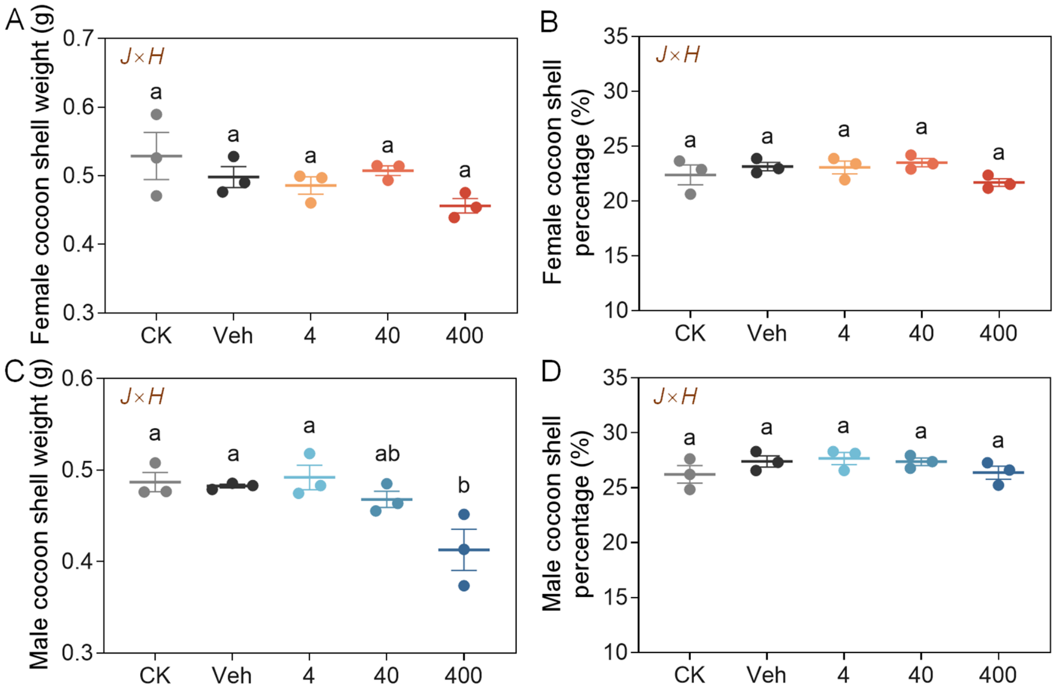

Figure S4. Effects of dietary 3-SeU-Ind supplementation on cocoon production traits in the *J×H* strain. (A & B) Cocoon shell weight (A) and cocoon shell ratio (B) of females. (C,D) Cocoon shell weight (C) and cocoon shell ratio (D) of males. Data are mean ± SD; *n* =3 biological replicates (15 individuals each) for (A–D). The different lower case letters show significant differences (P < 0.05).

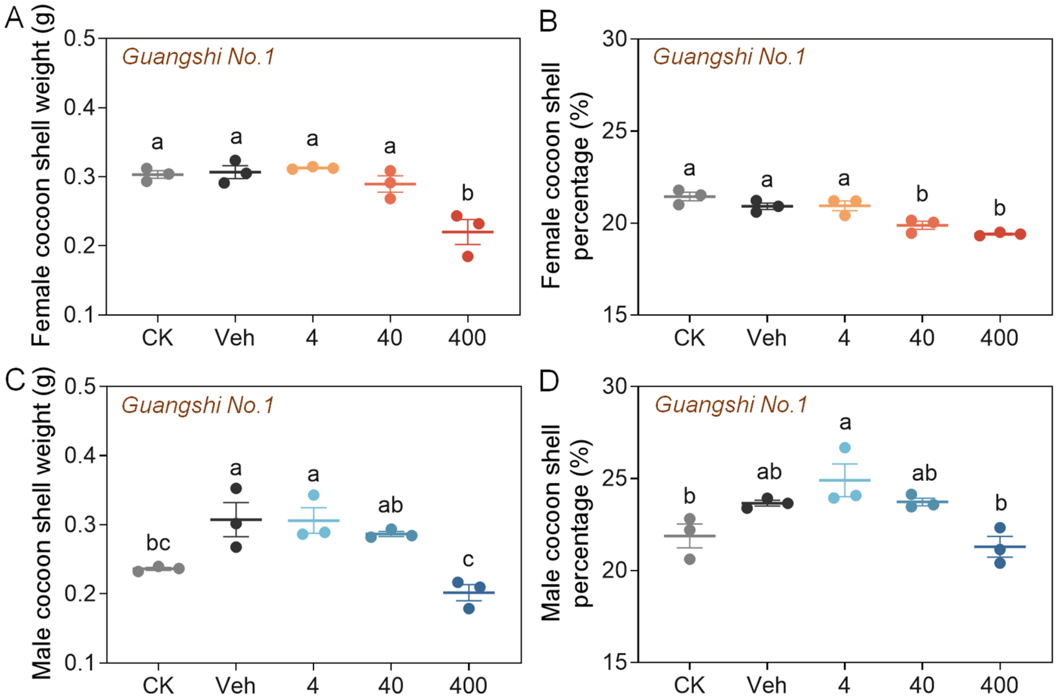

Figure S5. Effects of dietary 3-SeU-Ind supplementation on cocoon production traits in the *Guangshi NO.1* silkworm strain. (A & B) Cocoon shell weight (A) and cocoon shell ratio (B) of females. (C,D) Cocoon shell weight (C) and cocoon shell ratio (D) of males. Data are mean ± SD; *n* =3 biological replicates (15 individuals each) for (A–D). The different lower case letters show significant differences (P < 0.05).

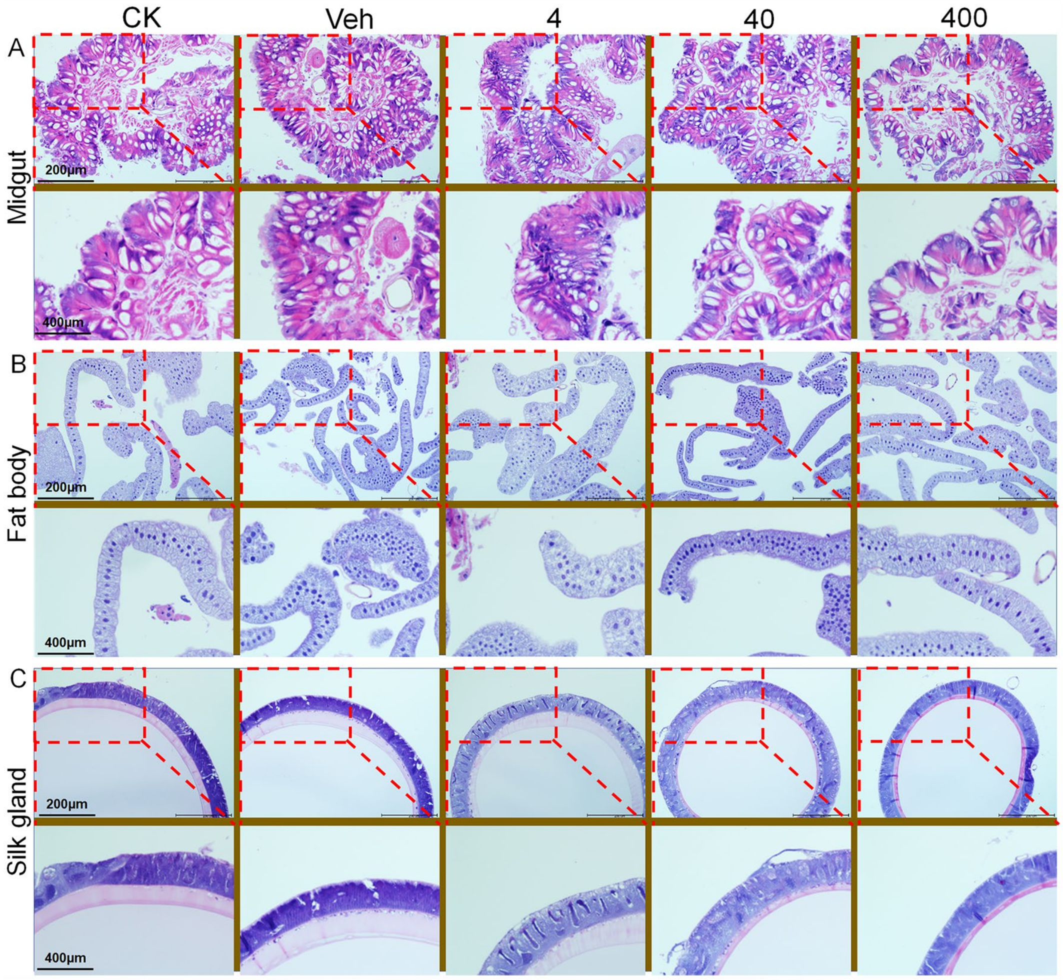

Figure S6. Effects of dietary 3-SeU-Ind supplementation on the midgut, silk gland, and fat body tissues of the *Dazao* strain. (A−C) Representative Hematoxylin-Eosin staining of the midgut (A), fat body (B), and silk gland (C). *n* =1 individual.

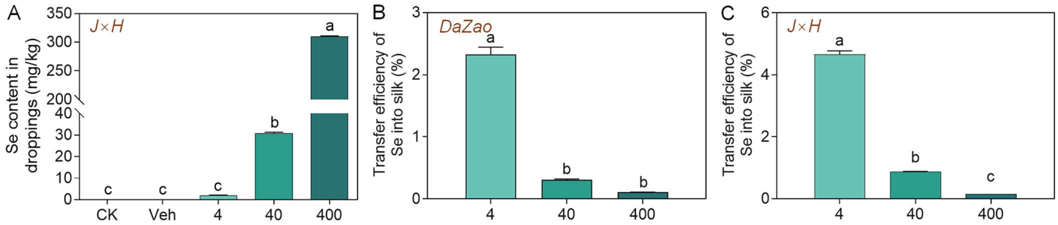

Figure S7. Se content in silkworm droppings and its transfer efficiency to silk following dietary 3-SeU-Ind supplementation. (A) Selenium content in the feces of the J×H strain. (B,C) Transfer efficiency of selenium to silk in the *Dazao* (B) and *J×H* (C) strains. Data are mean ± SD; *n* = 3 biological replicates (10–15 individuals each) for A; *n* = 3 biological replicates (3 individuals each) for (B,C). The different lower case letters show significant differences ($P < 0.05$).

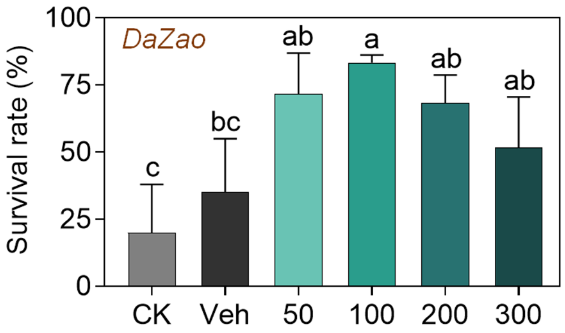

Figure S8. Effects of dietary 3-SeU-Ind supplementation on the survival rate of 5L *Dazao* larvae after 96 h of 34℃ heat stress. Data are mean ± SD; *n* = 3 biological replicates (30 individuals each). The different lower case letters show significant differences ($P < 0.05$).

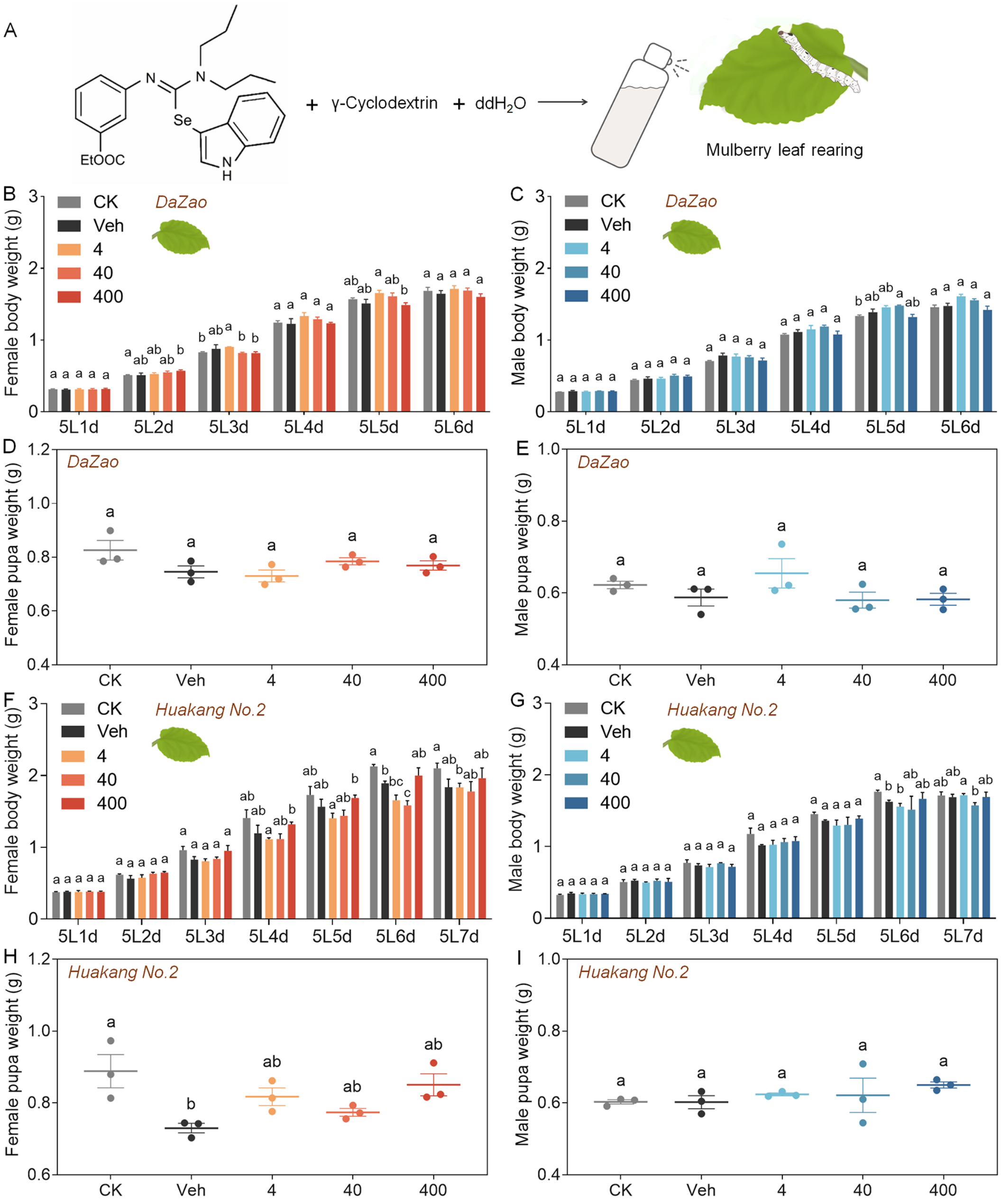

Figure S9. Impact of dietary supplementation with slightly modified 3-SeU-Ind on the body weight of male and female 5L silkworm larvae. (A) Molecular structure of slightly

modified 3-SeU-Ind and the experimental design for dietary supplementation in silkworms. (B,C) Changes in female (B) and male (C) body weight during the 5L larval stage of the *Dazao*. (D,E) Weights of female (D) and male (E) pupae from the *Dazao*. (F,G) Changes in female (F) and male (G) body weight during the 5L larval stage of the *Huakang NO.2*. (H,I) Weights of female (H) and male (I) pupae from the *Huakang NO.2*. Data are mean ± SD; n = 3 biological replicates (15 individuals each) for B−I. The different lower case letters show significant differences ($P < 0.05$).

Table S1. Formula for compound feed for silkworms

Component	Content (%)
Mulberry leaf powder	38.0
Soybean meal powder	42.4
Corn starch	11.2
Multivitamin	3.0
Others	5.4

Table S2. Primer Sequences

**Gene Names**	**Primer Sequences （5′→3′）**
BmRp49	F: CAGGCGGTTCAAGGGTCAATAC<br>R: TACGGAATCCATTTGGGAGCAT
BmCAT	F: AATGTCGGCGGAGATGTAGACC<br>R: GCAGCAGCATCCTTGAGGTGAT
BmTPX	F: ACTACGGAGTGCTGGACG<br>R: TCGGGCTTGATGGTCTT
BmMn-SOD	F: GATTCGAGTTGCCGGTGCTTCT<br>R: GCTTGTGCCTGTGCGAGTTTCT
BmGpx	F: CAAGAGCCTGGCAATCCAGA<br>R: CAAGGGTGCCTCCTTGCTTA
BmTrxR1	F: GTGTTCACGCCGTTAGAGTA<br>R: GGTAGCAGTTGCGGATGT
BmCncC	F: CCGGAAACGGAAACTGGA<br>R: CGGTATAGGGCCGCAAAC
BmSer-1	F: CTTGCCAGGAATCTAGACCGA<br>R: TGCACTTCCACCGACGAGA
BmSer-2	F: AAAAGTCCAGTTCTCGAGGA<br>R: TTGGAGCTCTTTCTAGACGA
BmSer-3	F: CGCTGCGGGTACAGGTAT<br>R: GCACTTCCACCGACGAGA
BmFib-H	F: ACAAGGTGCAGGAAGTGC<br>R: AGCAATTCACACAAGGCAGT
BmFib-L	F: CCGGAGGTGGAAGAATCTAT<br>R: GGTTATGTAGGCAGCGATGT
BmP25	F: CCCTGCTACTTGGACGATT<br>R: GATTATGGTCGACGTAGGTG

Table S3. Concentrations of various amino acids in hemolymph at 48 h after dietary 3-SeU-Ind supplementation.

	CK(μg/mL)	CD(μg/mL)	4(μg/mL)	40(μg/mL)	400(μg/mL)
Asp	13.92±0.63	31.04±1.13	24.10±2.61	24.18±5.92	34.50±2.18
Glu	43.19±4.78	60.59±9.28	51.38±5.29	47.04±4.42	50.07±0.89
Ser	881.41±7.86	928.27±34.55	1045.05±207.36	939.39±149.82	957.08±16.81
Gly	257.31±13.28	368.06±21.13	283.29±68.43	267.12±37.47	238.25±22.42
His	2182.92±63.08	2590.44±133.28	2358.72±78.59	2048.78±32.01	2184.38±174.13
Arg	326.75±18.93	552.34±37.05	345.70±23.94	351.78±53.32	278.34±43.48
Thr	329.12±36.68	491.15±53.37	321.70±57.93	329.47±20.62	297.18±36.54
Ala	339.11±23.98	409.47±15.15	296.59±72.45	288.70±53.09	270.25±24.60
Pro	182.44±26.80	336.05±27.37	170.19±16.64	200.96±25.87	163.33±17.95
Tyr	26.18±0.92	25.63±0.56	23.69±2.87	24.98±1.10	25.12±2.10
Val	248.36±50.94	299.65±25.24	197.68±56.89	353.92±233.05	175.11±13.07
Met	630.26±208.67	625.96±178.00	1352.06±73.83	1348.31±69.14	1309.68±52.82
Cys	2.12±0.39	3.92±0.85	2.91±0.38	3.34±0.24	2.43±0.79
Ile	101.70±1.93	144.38±8.96	85.64±7.30	95.23±10.28	78.70±12.95
Leu	138.95±3.83	173.62±12.61	107.19±7.15	117.45±24.48	96.47±30.17
Phe	136.12±3.23	140.65±12.62	110.70±8.05	116.60±9.28	120.63±5.12
